# Supplementary material for: The mechanism of DNA unwinding by the eukaryotic replicative helicase
Source: Nat Commun. 2019 May 14;10:2159. doi: 10.1038/s41467-019-09896-2 (PMC6517413; doi:10.1038/s41467-019-09896-2)
Supplement: Supplementary file 1 — Supplementary Information [file 41467_2019_9896_MOESM1_ESM.pdf]

Supplementary Information

The mechanism of DNA unwinding by the eukaryotic replicative helicase

Burnham et al.

Supplementary Figures

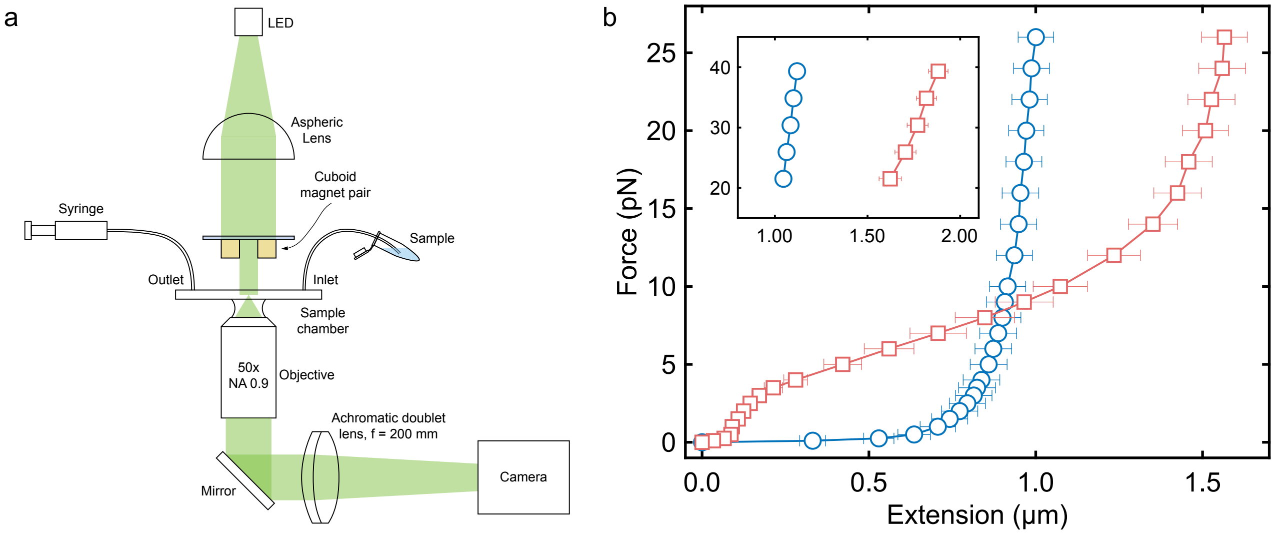

Supplementary Fig. 1 | Overview illustration of the magnetic tweezers microscope and force-extension curves for double-stranded (blue circles) and single-stranded (red squares) DNA. a, A brightfield microscope is constructed with illumination from an LED that is collimated with an aspheric lens, to pass through the gap formed by the magnet cube pair, onto the sample. The sample is imaged with a Nikon 50x NA 0.9 objective lens via an f = 200 mm tube lens onto a Falcon2 12M camera. Sample is drawn in through the inlet via a syringe fixed to the outlet. Further details in Methods. **b**, Force-extension curve for the 2.7 kb CMG unwinding template demonstrating the extension differences for varying forces using a magnet pair with a 1.5 mm gap between them. We take advantage of forces greater than ~ 9 pN to observe unwinding in real-time as the DNA becomes more extended upon conversion from ds- (blue circles) to ssDNA (red squares). Inset shows higher force curves using a magnet pair with gap of 0.5 mm. At 20, 30 and 39 pN the extension changes are 0.20 ± 0.03 $nmbp^{-1}$, 0.25 ± 0.02 $nmbp^{-1}$, and 0.28 ± 0.02 $nmbp^{-1}$, respectively. Error bars are standard error of the mean. For 0 to 20 pN force n = 12, for inset ssDNA n = 12, and inset dsDNA n = 39.

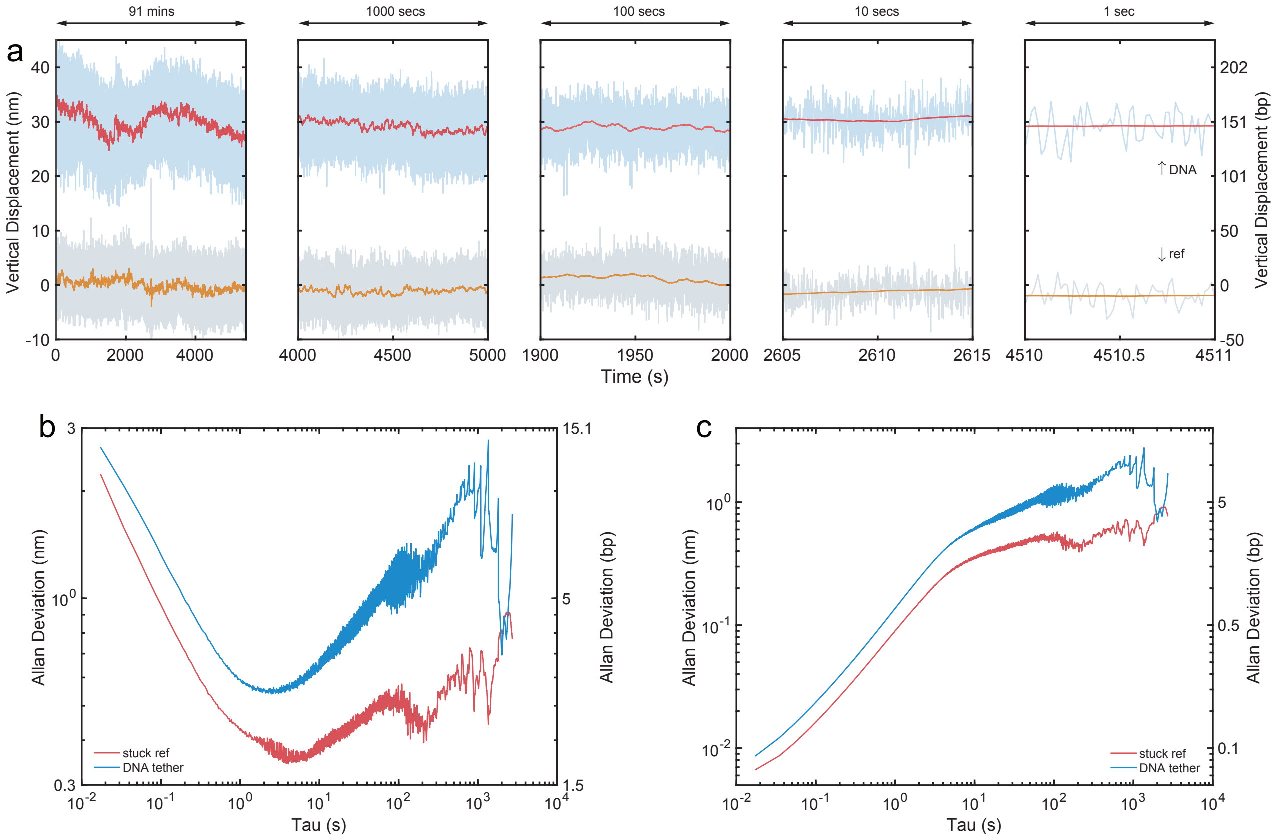

Supplementary Fig. 2 | Precision of microsphere vertical position measurements. Measurements of the vertical position of a reference microsphere, stuck on a glass coverslip, and a microsphere tethered to the glass surface, via an enzyme free 2.7 kb DNA molecule, were made for 91 mins. All microsphere trajectories were measured at 20 pN (noisiest force employed) and have been corrected through the subtraction of the mean of two reference microsphere trajectories. The standard deviations of the 0.17 Hz low pass filtered data are 1.2 ± 0.3 nm and 3.2 ± 1.2 nm (n = 3) for the reference and tethered microspheres, respectively. **a**, Example of DNA tethered and coverslip stuck microsphere vertical position trajectory at various timescales. The vertical displacement on the right hand axis uses the nanometre to base pairs conversion factor as used for a 20 pN unwinding experiment. The DNA tethered microsphere trajectory is displaced by 30 nm for clarity. The grey and light blue lines are trajectories at 58 Hz and the orange and red are trajectories low-pass filtered to 0.17 Hz. **b**, A more informative approach is to calculate the Allan deviation that describes the noise as a function of timescale [1,2]. Mean Allan deviation of 3 DNA tethered microspheres (top blue line) and 3 reference microspheres (bottom red line). As expected the reference microsphere obtains better precision over all timescales. The DNA tether obtains precision sufficient enough to detect the signals we report. The Allan deviation on the right hand axis uses the nanometre to base pairs conversion factor as used for a 20 pN unwinding experiment. **c.** Although not strictly an Allan deviation we also plot the Allan deviation of the same trajectories after low-pass filtering to 0.17 Hz. This is informative on the effects of filtering and the effective reduction in noise. The Allan deviation on the right hand axis uses the nanometre to base pairs conversion factor as used for a 20 pN unwinding experiment.

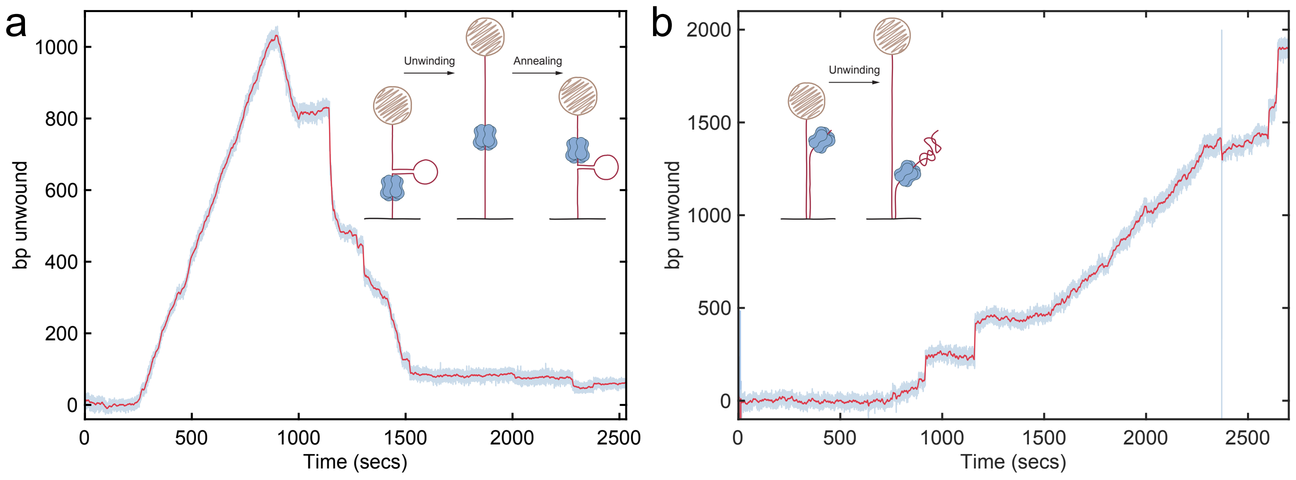

Supplementary Fig. 3 Typical trajectories of single SV40 large T antigen helicases unwinding DNA. a, Unwinding of a 1 kb DNA hairpin template. The trajectory closely resembles those observed for other homohexameric ring helicases such as DnaB [3] and T4 gp41 [4], except for the longer timescale of unwinding, which is expected due to 10 fold slower eukaryotic replication rates [5]. **b**, Unwinding of a 2711 bp template duplex DNA fork template. This trajectory closely resembles those observed for DnaB [3] and T4 gp41 [6] but over longer timescales and also similar experiments observing bi-directional unwinding by large T antigen [7]. The light blue lines are trajectories at 58 Hz and the red are trajectories low-pass filtered to 0.17 Hz.

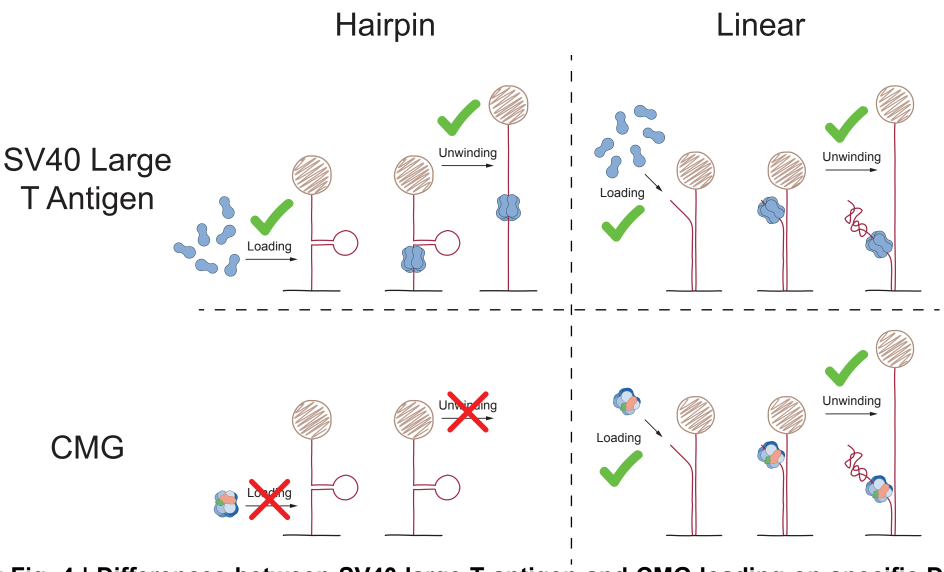

Supplementary Fig. 4 | Differences between SV40 large T antigen and CMG loading on specific DNA constructs. Top panels) SV40 large T antigen can bind to and form a hexamer on both a hairpin and linear DNA template, where a 3’ flap is and is not available, respectively. **Bottom panels)** Due to the tightly regulated process of eukaryotic helicase CMG loading, Cdc45 and GINS effectively close the ring structure of the heterohexameric Mcm2-7. This prevents CMG binding around a hairpin strand as there is no free 3’ end but it can ‘thread’ onto a free 3’ end contained within the linear substrate.

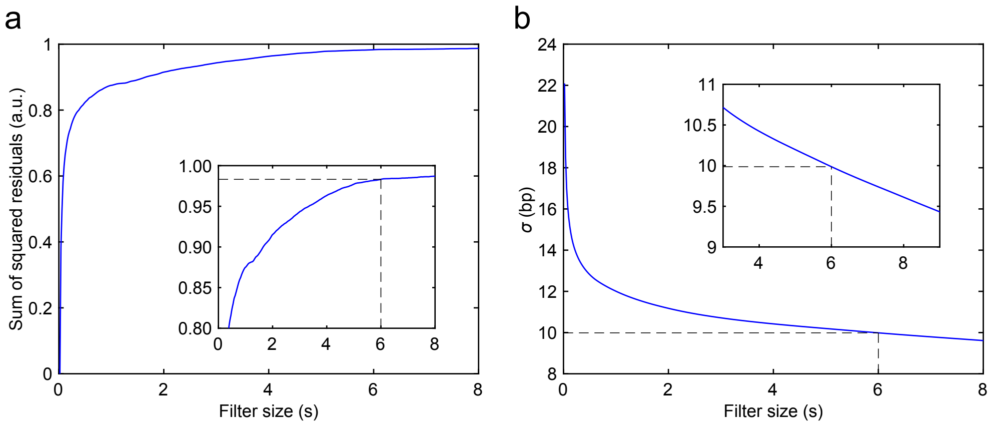

Supplementary Fig. 5 | Determining filter and passage interval size. a, Squared residual as a function of moving mean filter size. A filter size is chosen such that a larger filter does not increase the magnitude of the residuals between raw and filtered data. Inset) Portion of same plot with a 6 s filter chosen. **b**, Standard deviation, σ, of an 80 s enzyme free trajectory at 20 pN as a function of filter size. Given the chosen 6s filter size corresponds to a standard deviation of 10 bp the passage interval is set to 20 bp = 2σ.

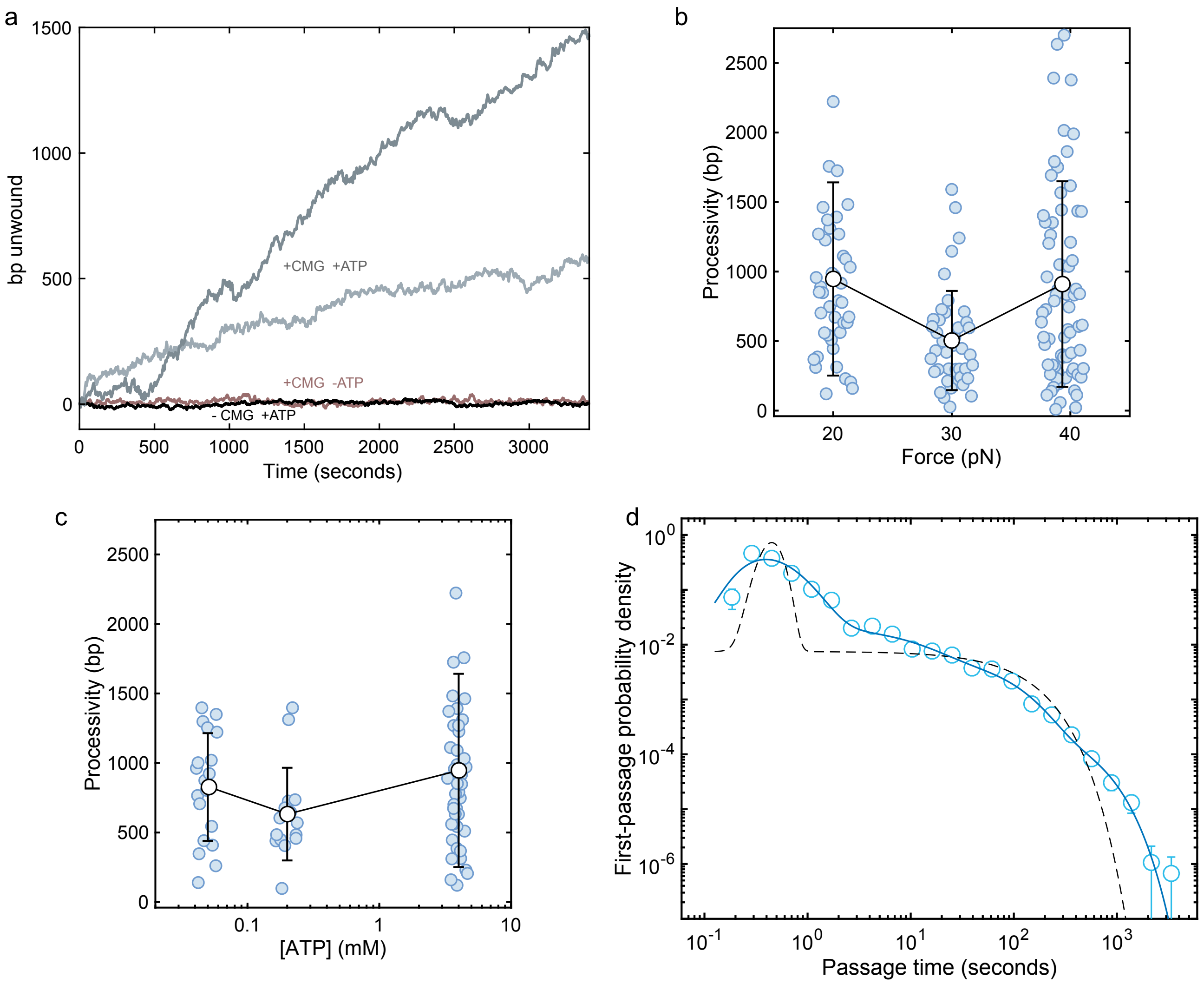

Supplementary Fig. 6 | Example unwinding and control traces; unwinding processivities for CMG on the DNA unwinding template; and comparison of fitting data with unidirectional and biased random walk models. a, Example unwinding traces (20 pN, 4 mM ATP) with both CMG and ATP included. The standard protocol is followed with the exclusion of either CMG or ATP. As our experimental assay does not wait for the CMG unwinding reaction to 'complete' we do not know if we have reached the limit of CMG processivity. One may imagine the processivity measured will be linear unwinding rate dependent. Therefore we only quote in the main text the mean processivity measured as a lower limit on CMG processivity as 827 ± 642 bp (n = 197). **b**, The mean and standard deviation for 20 pN, 30 pN, and 39.3 pN are 947 ± 695 bp (n = 46), 504 ± 357 bp (n = 42) and 910 ± 740 bp (n = 75) respectively. Error bars are standard deviation **c**, For ATP concentrations of 0.05 mM, 0.2 mM, and 4 mM are 827 ± 387 bp (n = 15), 632 ± 333 bp (n = 19), and 947 ± 695 bp (n = 46), respectively. Error bars are standard deviation **d**, A unidirectional helicase would exhibit stochastic behaviour resulting in a first-passage time described by a gamma function [8]. Here, an example experimental first-passage time probability distribution (blue circles), at 20 pN and 4 mM ATP, is fit via maximum likelihood estimation. Using the BIC as a metric such a gamma function best describes the data with the inclusion of a single pause (dashed black line). As comparison our favoured model of a biased random walk with multiple pauses is also fit and plot (blue line).The BIC for a unidirectional helicase with single pause is much greater than that calculated for a biased random walker with three pauses, thus quantitatively supporting the biased random walk model over the unidirectional model. Error bars are standard deviations from 1000 bootstraps.

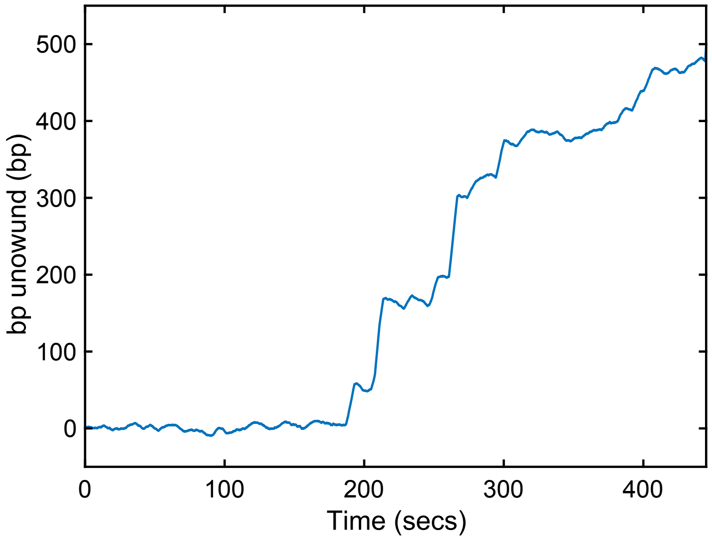

Supplementary Fig. 7 | A plot showing the start of CMG unwinding a linear DNA substrate with 3' flap after addition of ATP before time zero. Unwinding predominantly starts during ATP addition. As standard in the field, tracking of the motor protein position is lost during this addition due to flow in the sample chamber, so we cannot display it for all traces. However, we display here an example trajectory, at 20 pN and 4 mM ATP, demonstrating that after ATP addition there is a period of no unwinding before the helicase begins unwinding.

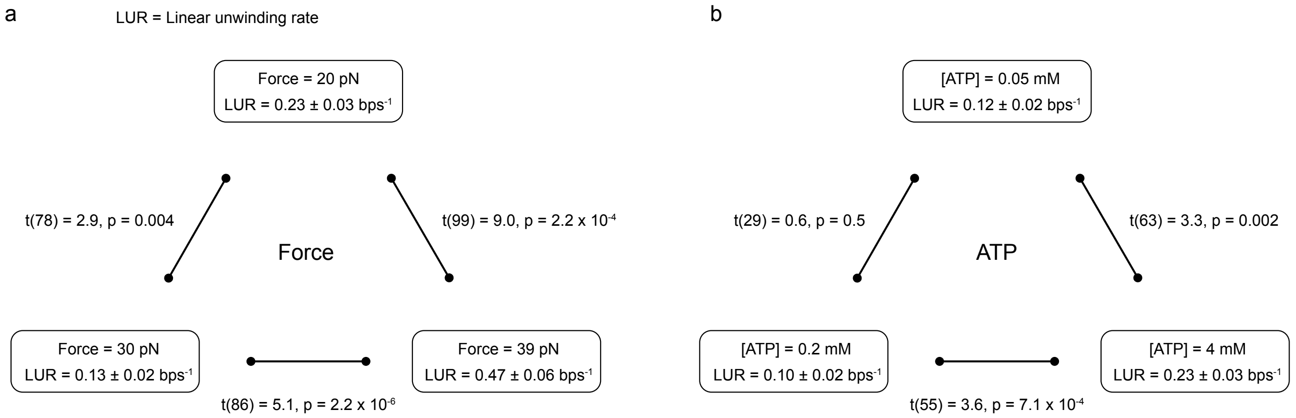

Supplementary Fig. 8 Two-sided Welch's t-test statistics comparing each distribution to others for figures 2a and 2b in a and b, respectively.

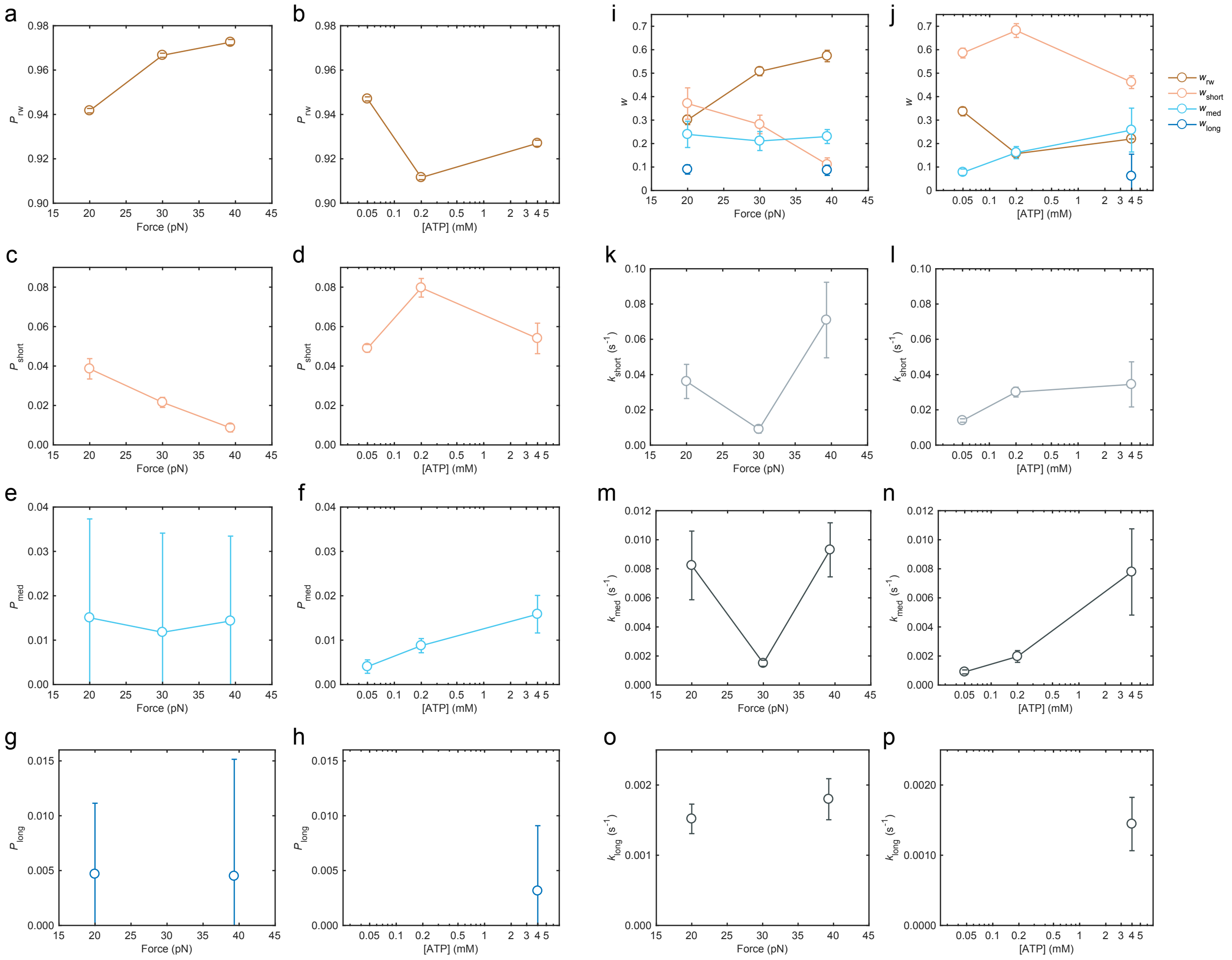

Supplementary Fig. 9 | Probabilities of entering the reported kinetic states on a single nucleotide basis; proportion of first-passage times accounted for by the states described in the main text; and exit rates from pause states identified in the main text. This data is from Fig. 4a and 4d in the main text but plotted here individually on linear scales for clarity. Probability of entering, as a function of force or ATP concentration, **a**, and **b**, a random walk, **c**, and **d**, a short pause, **e**, and **f**, a medium pause, and **g**, and **h**, a long timescale pause. Proportion of first-passage times accounted for by the biased random walk, w_{rw}, short timescale pausing, w_{short}, medium timescale pausing, w_{med}, and long timescale pausing, w_{long} as a function of **i**, force and **j**, ATP concentration. Rate of exit from short timescale pauses, k_{short}, as a function of **k**, force and **l**, ATP concentration. Rate of exit from medium timescale pauses, k_{med}, as a function of **m**, force and **n**, ATP concentration. Rate of exit from long timescale pauses, k_{long}, as a function of **o**, force and **p**, ATP concentration. All error bars are standard deviations from 1000 bootstraps.

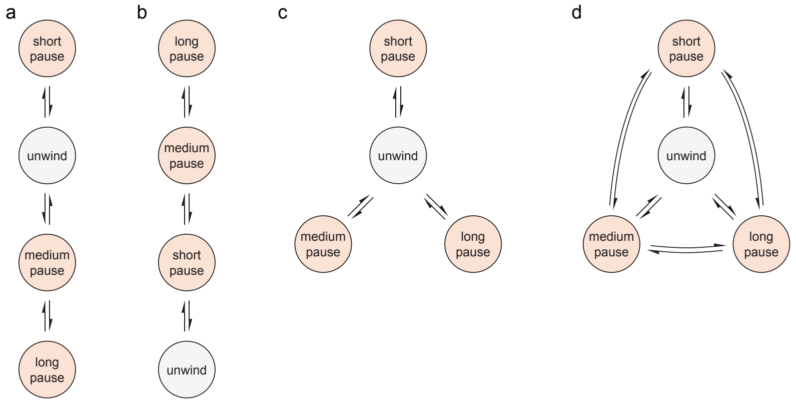

Supplementary Fig. 10 | Alternate kinetic schemes. Here unwind refers to the biased random walk of DNA unwinding and each scheme has what we term short, medium and long timescale non-productive pauses. **a**, The short pause is separated from medium and long. **b**, The pauses are interconnected. **c**, The scheme we have used in the main manuscript. **d**, A cyclic kinetic scheme. This list is by no means exhaustive and displays only some examples.

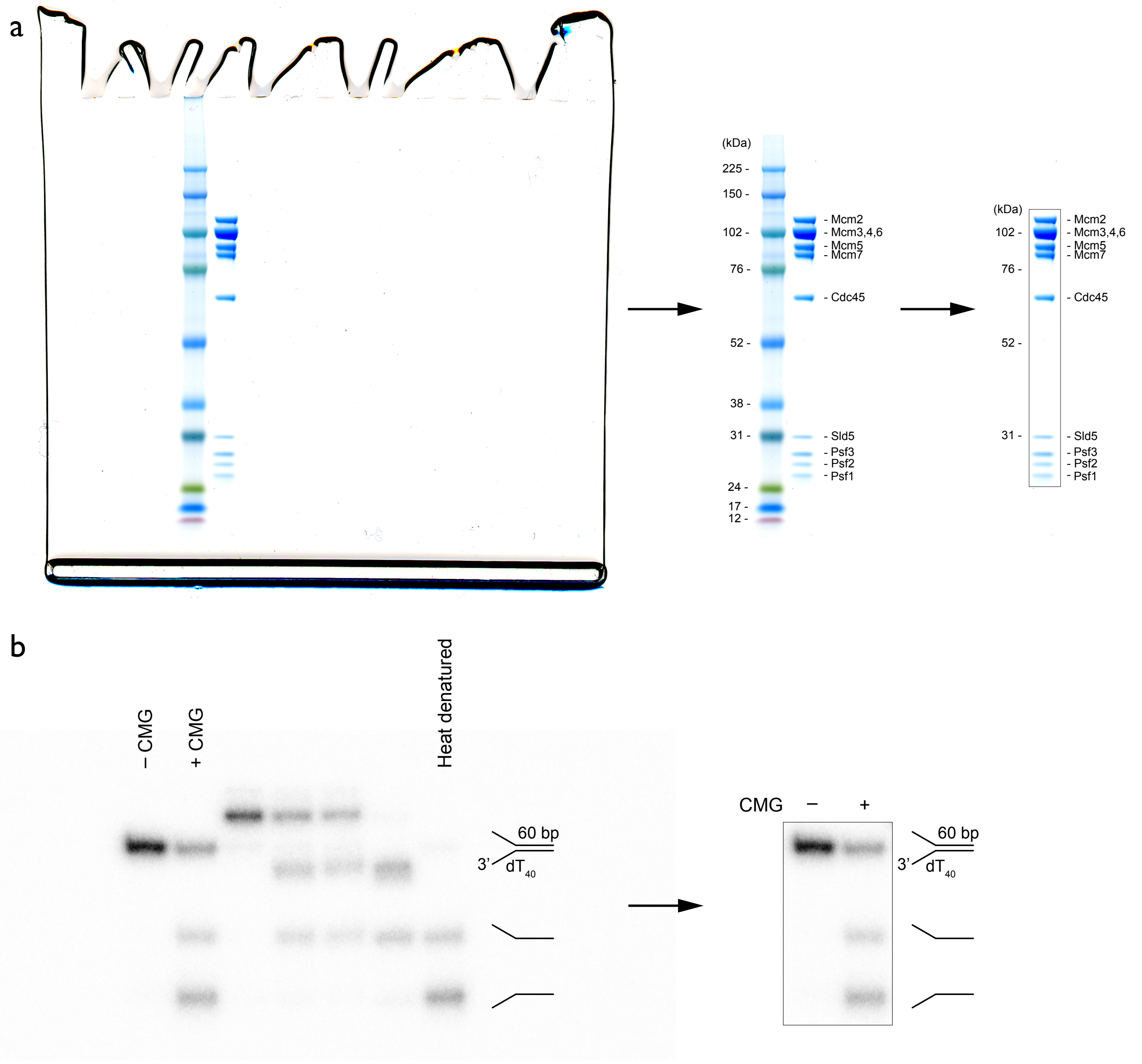

Supplementary Fig. 11 | Original gel images used for Fig. 1. a, Gel used for Fig. 1a. Coomassie stained 4-12% SDS-PAGE gel of purified *Dm*CMG. Sld5, Psf1-3 forms GINS. Marker lane is Amersham ECL Rainbow Marker - Full range (Sigma, GERPN800E) **b**, Gel used for Fig. 1b. Example of bulk unwinding of duplex DNA, radiolabelled at 5' ends, by CMG. The 60 bp duplex with 40 nt 3' polyT tail is unwound into the two single strands. ssDNA is identified by heat denaturation of duplex. No marker is included as the readout was separation of ssDNA from dsDNA; the absolute size is not relevant. The unlabelled lanes are not relevant for this study.

ATP (mM)	0.05	0.2	4	4	4
Force (pN)	20	20	20	30	39.3
$n_{molecules}$	15	19	46	42	75
Linear unwinding rate (mean ± sem)	0.12 ± 0.02	0.10 ± 0.02	0.23 ± 0.03	0.13 ± 0.02	0.47 ± 0.06
$n_{passage\ times}$	778	527	2034	865	2515
r_f (s^{-1})	239 ± 23	113 ± 19	141 ± 10	195 ± 14	226 ± 9
r_b (s^{-1})	218 ± 24	81 ± 19	121 ± 11	177 ± 14	208 ± 11
k_{short} (s^{-1})	0.014 ± 0.001	0.030 ± 0.003	0.036 ± 0.00968	0.0090 ± 0.0022	0.071 ± 0.021
k_{med} (s^{-1})	0.0009 ± 0.0001	0.0019 ± 0.0004	0.0082 ± 0.0024	0.0015 ± 0.00021	0.0093 ± 0.0018
k_{long} (s^{-1})	-	-	0.0015 ± 0.0002	-	0.0018 ± 0.0003
w_{rw}	0.34 ± 0.02	0.16 ± 0.02	0.30 ± 0.02	0.51 ± 0.02	0.57 ± 0.02
w_{short}	0.59 ± 0.02	0.68 ± 0.03	0.37 ± 0.07	0.28 ± 0.04	0.11 ± 0.03
w_{med}	0.08 ± 0.01	0.16 ± 0.03	0.24 ± 0.06	0.21 ± 0.04	0.23 ± 0.03
w_{long}	-	-	0.09 ± 0.02	-	0.09 ± 0.02
P_{rw}	0.9470 ± 0.0009	0.912± 0.001	0.942 ± 0.001	0.9667 ± 0.0009	0.973 ± 0.001
P_{short}	0.049 ± 0.002	0.080 ± 0.005	0.039 ± 0.005	0.022 ± 0.003	0.009 ± 0.002
P_{med}	0.004 ± 0.002	0.009 ± 0.002	0.015 ± 0.022	0.012 ± 0.022	0.014 ± 0.019
P_{long}	-	-	0.005 ± 0.007	-	0.005 ± 0.011
v_{mean} (bps^{-1})	21 ± 34	32 ± 27	21 ± 15	18 ± 13	19 ± 16
D_{eff}	229 ± 17	97 ± 14	131 ± 8	186 ± 7	217 ± 8

Supplementary Table 1 | Parameters extracted from experimental data and associated errors and statistics. All uncertainties are standard deviation unless stated otherwise.

Oligo	Sequence	Supplier
1	ATGCCGGGAGCAGACAAGCCCGTC	Eurofins Genomics
2	5’ Phosphorylation AGGTCGCCGCCCGGAAGAGCAGCTGGCACGACAGGTTTCCCG	IDT
3	AGGTCGCCGCCC	Eurofins Genomics
4	5’ Phosphorylation GGGCGGCGACCTGGAAGAGCAGCTGGCACGACAGGTTTCCCG	IDT
5	GGGCGGCGACCT	IDT
6	AGAGCTCCTCAGCCCGGCAACAATTAATAGACTGGATG	IDT
7	TAGCGCCTCAGCGAAGCTAGAGTAAGTAGTTCGCC	IDT
8	TTTTTTTGGAGCTCT	IDT
9	TTTTTTGGCGCTA	IDT
10	ATGTAAGCCTCAGCAGCTGGCACGACAGGTTTCCCG	IDT
11	GGCTTACATTTTTTT	IDT
12	GGCAAGAGCAACTCGGTCGCCGCATACACTATTCTCAGAATGACTTGGTTATGTAAGCC	IDT
13	5’ Phosphorylation<br>AACCAAGTCATTCTGAGAATAGTGTATGCGGCGACCGAGTTGCTCTTGCCGGCAGGCAG<br>GCAGGCAGGCAGGCAGGCAGGCAGGCATGCTCTTTACAACCGGTAGACTGCTTC<br>AGGGAACGATGTGCTGTGTACAGAGCTCC	IDT
14	5’ Phosphorylation<br>GTACACAGCACATCGTTCCCTGAAGCAGTCTACCGGTTGTAAAGAGCATTTTTTTTTTTTT<br>TTTTTTTTTTTTTTTTTTTTTTTTTTTTTTTTTTTTTTTTTTTTTTTTTTTTTTTTT	IDT
15	5' phosphorylation<br>ATGCTCTTTACAACCGGTAGACTGCTTCAGGGAACGATGTGCTGTGTACGCCAGAAGGT<br>AAGCCCTCCATCGTTCAGGCAACTATGGATGAACGAATGTAAGCC	IDT
16	GACAGAGTACACAGCACATCGTTCCCTGAAGCAGTCTACCGGTTGTAAAGAGCATTAGC<br>GCC	IDT
17	5’ Phosphorylation TCGTTCATCCATAGTTGCCTGAACGATGGAGGGCTTACCTTCTGGC	IDT
18	GGCTCGTTTTACAACGTCGTGCTGAGGTGATATCTGCTGAGGCAATGGGAATTCGCCAA<br>CCTTTTTTTTTTTTTTTTTTTTTTTTTTTTTTTTTTTTTTTT	IDT
19	GGCAGGCAGGCAGGCAGGCAGGCAGGCAGGCAGGCAGGCAGGTTGGCGAATTCCCAT<br>TGCCTCAGCAGATATCACCTCAGCACGACGTTGTAAAACGAG	IDT
20	AGAGCTCCTCAGCGTCTCATGAGCGGATACATA	IDT
21	TAGCGCCTCAGCACGCCAGCAACGCGG	IDT
22	5’ Phosphorylation<br>AACCAAGTCATTCTGAGAATAGTGTATGCGGCGACCGAGTTGCTCTTGCCATGCTCTTTA<br>CAACCGGTAGACTGCTTCAGGGAACGATGTGCTGTGTACAGAGCTCC	IDT
23	5’ Phosphorylation<br>GTACACAGCACATCGTTCCCTGAAGCAGTCTACCGGTTGTAAAGAGCATTTTTTTTTTTTT<br>TTTTTTTTTTTTTTTTTTTTTTTTTTTTTTTTTTTTTTGCCAGAAGGTAAGCCCTCCATCGTT<br>CAGGCAACTATGGATGAACGAATGTAAGCC	IDT
24	5’ Phosphorylation AGCGAAAGCTTAGCGCC	IDT

Supplementary Table 2 | Oligos used to make the DNA templates for calibration and unwinding.

Supplementary Notes

Supplementary Note 1

Using forces between 20 and 40 pN prevent re-annealing behind the helicase [9] and permits spontaneous re-annealing from the ss-dsDNA junction [10], ahead of the helicase.

Prevention of annealing behind helicase at high force

To re-anneal partially unwound DNA behind the helicase the leading, tracked strand, must overcome an activation energy to match the stretched, excluded, lagging strand. Following Dessinges *et al.* [9] we can estimate the thermal fluctuation required to achieve this, $\Delta E_a = N.F.\partial L(F)$, where N is the helicase binding footprint, F is the applied force, and $\partial L(F)$ is the change in extension between CMG bound ssDNA and dsDNA. To calculate the change in extension of DNA within CMG to that of stretched ssDNA we note that the ssDNA within the central channel has a structural form similar to that of a single strand extracted from B-form dsDNA [11]. For CMG with a DNA footprint of $N \approx 34$ nt, a change in DNA extension of a single nucleotide gap between dsDNA to ssDNA at F = 20 pN is $\partial(20\ \text{pN})$ = 0.198 nm; giving ΔE_a = 29 $k_B T$, reducing the probability of hybridisation by 14 orders of magnitude. Therefore, DNA does not re-anneal behind the helicase even at the lowest force (20 pN) used in our experiments.

Supplementary Note 2

Upon analysis ~10% of all single DNA molecules show activity. Considering the Poisson distribution, we see the probability of a single DNA molecule being unwound by x helicases, $P_\mu(x)$, when the ratio of molecules with activity to total number of molecules is is;

$$P_\mu(x) = \mathrm{e}^{-\mu}\frac{\mu^x}{x!} \quad (1)$$

The probability for any given single DNA molecule to be unwound by x = 0, 1, 2, or 3 helicases is then;

$$P_{0.1}(0) = \mathrm{e}^{-0.1}\frac{0.1^0}{0!} = e^{0.1} = 0.90 \quad (2)$$

$$P_{0.1}(1) = \mathrm{e}^{-0.1}\frac{0.1^1}{1!} = 0.1e^{0.1} = 0.090 \quad (3)$$

$$P_{0.1}(2) = \mathrm{e}^{-0.1}\frac{0.1^2}{2!} = \frac{1}{2}0.01e^{0.1} = 0.0045 \quad (4)$$

$$P_{0.1}(3) = \mathrm{e}^{-0.1}\frac{0.1^3}{3!} = \frac{1}{6}0.001e^{0.1} = 0.00015 \quad (5)$$

Thus, the likelihood of a single DNA molecule being unwound by 2 or more helicases is 1%. Of the molecules that exhibit activity, the likelihood of 2 or more helicases being responsible is 10% . Notably in our assay there are unlikely to be 2 helicases per DNA molecule due to the length of the 3' flap and pre-loading of CMG. This calculated value can thus be treated as an upper limit.

Supplementary Note 3

Notes on supplementary figure 4

We can observe SV40 large T-antigen unwinding both hairpin and linear substrates but cannot observe CMG unwinding a hairpin. This is not due to the sensitivity of the assay, but stems from the nature of the helicase loading mechanism and DNA template geometry.

SV40 large T antigen can, demonstrably, load onto DNA without a free end and onto origin DNA without additional factors [12]. This is likely due to the homohexameric construction and simpler DNA loading mechanism for this helicase, for example, the binding of monomers to DNA to form the hexamer (Supplementary Figure 4, top panels) [13]. CMG loading is much more tightly regulated [14], and pre-formed CMG complex cannot load onto DNA without a free 3’ end unless Mcm10 is present [15]. Indeed, bulk biochemical assays are performed on fork substrates or substrates with a 3’ free end to allow loading [16]. We believe that in our assay we can observe unwinding on the linear 2.7 kb duplex DNA substrate because the closed CMG ring can thread onto the 3’ free end (Supplementary Figure 4, bottom right panel) as is observed in structural work [17]. We do not observe unwinding when using a hairpin because it has no free end for CMG to thread onto, thus preventing loading and subsequently unwinding (Supplementary Figure 4, bottom left panel). Without the tightly regulated loading pathway and associated additional factors CMG is not loaded onto DNA without a free end.

Supplementary Methods

Buffers

Blocking buffer - kept in 4ºC

20mM Tris-HCl pH 7.5, 2mM EDTA, 50mM NaCl, 0.05mg/mL BSA, 0.1% (v/v) Tween.

Binding buffer - kept at room temperature

20mM Tris-HCl pH 7.5, 5 mM EDTA, 20 mM NaCl.

CMG Loading Buffer (DTT added shortly before use)

25 mM HEPES pH 7.5, 5 mM NaCl, 10 mM MgOAc, 5 mM DTT, 0.05 mg/mL BSA, 4 mM ATPgS.

CMG Running Buffer

25 mM HEPES pH 7.5, 5 mM NaCl, 10 mM MgOAc, 5 mM DTT, 0.05 mg/mL BSA, [Desired Conc] ATP, 20 mM Phosphocreatine, 1 ug/ml Creatine phosphokinase.

Re-suspension buffer C

25 mM Hepes pH 7.6, 0.02% Tween-20, 10% glycerol, 1 mM EDTA, 1mM EGTA, 15mM KCl, 2 mM $MgCl_2$, 2 mM 2-mercapotoethanol, 0.4mM PMSF, (2 tablets/100 ml) complete protease inhibitors cocktail from Roche diagnostics (Roche, 5056489001).

Buffer C

25 mM HEPES (pH 7.5), 0.02% Tween-20, 10% glycerol, 1 mM EDTA, 1 mM EGTA.

Buffer C-100

25 mM HEPES (pH 7.5), 0.02% Tween-20, 10% glycerol, 1 mM EDTA, 1 mM EGTA, 100 mM KCl,_1 mM DTT.

Buffer C-150-no Tween

25 mM HEPES (pH 7.5), 10% glycerol, 1 mM EDTA, 1 mM EGTA, 150 mM KCl, 1 mM DTT.

Buffer C-550

25 mM HEPES (pH 7.5), 0.02% Tween-20, 10% glycerol,1 mM EDTA, 1 mM EGTA, 550 mM KCl, 1 mM DTT.

Elution buffer

25 mM HEPES (pH 7.5), 0.02% Tween-20, 10% glycerol, 1mM EDTA, 1mM EGTA, 100 mM KCl, 1mM DTT, 1 tablet/10 ml protease inhibitor tablet.

Dialysis buffer

25 mM HEPES (pH 7.5),10% glycerol, 50 mM NaOAc,10 mM MgOAc, 1 mM DTT.

STE buffer

10 mM Tris-HCl, pH 8.0, 100 mM NaCl, 1 mM EDTA

L-Tag re-suspension buffer

20 mM Tris, pH 9.0, 300mM NaCl, 1mM EDTA, 10% glycerol, 0.5% NP-40, 0.1mM DTT

L-Tag Neutralisation buffer

100 mM Tris, pH 6.8, 300mM NaCl, 1mM EDTA, 10% glycerol, 0.5% NP-40, 0.1mM DTT

L-Tag Loading buffer

20 mM Tris, pH 8, 300mM NaCl, 1mM EDTA, 10% glycerol, 0.5% NP-40, 0.1mM DTT

L-Tag Wash buffer

50 mM Tris, pH 8.0, 1M NaCl, 1mM EDTA, 10% glycerol

L-Tag EG buffer

50 mM Tris, pH 8.5, 500mM NaCl, 1mM EDTA, 10% glycerol, 10% ethylene glycol

L-Tag Elution buffer

50 mM Tris, pH 8.5, 1M NaCl, 10mM MgCL2, 1mM EDTA, 10% glycerol, 55% ethylene glycol

L-Tag Dialysis buffer

20 mM Tris, pH 8.0, 10mM NaCl, 1mM EDTA, 50% glycerol, 1mM DTT

Supplementary References

1. Gibson, G. M., Leach, J., Keen, S., Wright, A. J. & Padgett, M. J. Measuring the accuracy of particle position and force in optical tweezers using high-speed video microscopy. *Opt. Express* 16, 14561 (2008).

2. Czerwinski, F., Richardson, A. C. & Oddershede, L. B. Quantifying noise in optical tweezers by Allan variance. Opt. Express 17, 13255–13269 (2009).

3. Ribeck, N., Kaplan, D. L., Bruck, I. & Saleh, O. A. DnaB Helicase Activity Is Modulated by DNA Geometry and Force. *Biophys. J.* 99, 2170–2179 (2010).

4. Lionnet, T., Spiering, M. M., Benkovic, S. J., Bensimon, D. & Croquette, V. Real-time observation of bacteriophage T4 gp41 helicase reveals an unwinding mechanism. *Proc. Natl. Acad. Sci.* 104, 19790–19795 (2007).

5. Raghuraman, M. K. Replication Dynamics of the Yeast Genome. *Science* 294, 115–121 (2001).

6. Ribeck, N. & Saleh, O. A. DNA Unwinding by Ring-Shaped T4 Helicase gp41 Is Hindered by Tension on the Occluded Strand. *PLoS ONE* 8, e79237 (2013).

7. Berghuis, B. A., Köber, M., van Laar, T. & Dekker, N. H. High-throughput, high-force probing of DNA-protein interactions with magnetic tweezers. *Methods* 105, 90–98 (2016).

8. Floyd, D. L., Harrison, S. C. & van Oijen, A. M. Analysis of Kinetic Intermediates in Single-Particle Dwell-Time Distributions. *Biophys. J.* 99, 360–366 (2010).

9. Dessinges, M.-N., Lionnet, T., Xi, X. G., Bensimon, D. & Croquette, V. Single-molecule assay reveals strand switching and enhanced processivity of UvrD. *Proc. Natl. Acad. Sci.* 101, 6439–6444 (2004).

10. Smith, S. B., Cui, Y. & Bustamante, C. Overstretching B-DNA: the elastic response of individual double-stranded and single-stranded DNA molecules. *Science* 271, 795–799 (1996).

11. Abid Ali, F. et al. Cryo-EM structures of the eukaryotic replicative helicase bound to a translocation substrate. *Nat. Commun.* 7, 10708 (2016).

12. Yardimci, H. et al. Bypass of a protein barrier by a replicative DNA helicase. *Nature* 492, 205–209 (2012).

13. Dean, F. B., Borowiec, J. A., Eki, T. & Hurwitz, J. The simian virus 40 T antigen double hexamer assembles around the DNA at the replication origin. *J. Biol. Chem.* 267, 14129–14137 (1992).

14. Douglas, M. E., Ali, F. A., Costa, A. & Diffley, J. F. X. The mechanism of eukaryotic CMG helicase activation. *Nature* (2018).

15. Wasserman, M. R., Schauer, G. D., O'Donnell, M. E. & Liu, S. Replisome preservation by a single-stranded DNA gate in the CMG helicase. Preprint at bioRxiv (2018). Preprint at https://www.biorxiv.org/content/10.1101/368472v1 (2018)

16. Langston, L. D. et al. Mcm10 promotes rapid isomerization of CMG-DNA for replisome bypass of lagging strand DNA blocks. *Elife* 6, (2017).

17. Georgescu, R. et al. Structure of eukaryotic CMG helicase at a replication fork and implications to replisome architecture and origin initiation. *Proc. Natl. Acad. Sci.* 114, E697–E706 (2017).
